# Genetic and Epigenetic Signatures for Cognitive Resilience in Neuropathologically Defined Alzheimer’s Disease Brains

**DOI:** 10.1002/alz.089466

**Published:** 2025-01-09

**Authors:** Xudong Han, Donghe Li, Daniel Goldstein, Nathan J Sahelijo, Lindsay A. Farrer, Thor D. Stein, Jesse Mez, Gyungah R Jun

**Affiliations:** ^1^ Boston University Chobanian & Avedisian School of Medicine, Boston, MA USA; ^2^ Boston University School of Public Health, Boston, MA USA; ^3^ VA Boston Healthcare System, Boston, MA USA; ^4^ Boston University Chronic Traumatic Encephalopathy Center, Boston University Chobanian & Avedisian School of Medicine, Boston, MA USA

## Abstract

**Background:**

Not all people with neuropathological evidence of Alzheimer’s disease (AD) manifest clinical symptoms in life (cognitive resilience). We aimed to identify genetic and epigenetic signatures of cognitive resilience, utilizing data from brain donors with neuropathological evidence of AD who were either symptomatic or asymptomatic in life.

**Method:**

Among brain donors with neuropathologically‐confirmed AD (364 asymptomatic/cognitively resilient and 490 symptomatic) from the Boston University AD Research Center, Framingham Heart Study—where we generated our own data—as well as the Religious Orders Study and Rush Memory and Aging Project, we utilized genome‐wide genetic array data, genome‐wide DNA methylation array data and RNA sequencing data. We conducted a genome‐wide association study (GWAS) and a methylation‐wide association study (MWAS) in each dataset and meta‐analyzed. Relationships between top‐ranked CpG sites, SNPs, and expression levels of the selected genes/isoforms were further evaluated.

**Result:**

Meta‐analysis of genome‐wide significant MWAS results revealed 3 CpG sites from 3 genes, ARHGAP22, UBAC2, and KANSL1 (top‐ranked= cg19832721: Log2 fold change [Log2FC]=‐0.12, P=2.5x10^‐8^). Meta‐analysis of GWAS findings identified significant ((P<1x10^‐5^) associations with 9 single nucleotide polymorphisms (SNPs) located in 9 different loci including APOE (top‐ranked=rs6917901 in TNFAIP3: odds ratio [OR]=0.53, 95% confidence interval [CI]=0.41‐0.67, P=3.8x10^‐7^). Expression of KANSL1 and its isoform ENST00000638269 were increased in symptomatic compared with cognitive resilient AD subjects (gene: Log2FC=0.082, P=5.95x10^‐6^; isoform: Log2FC=0.325, P=5.95x10^‐4^). The methylation level of cg19832721 was negatively correlated with expression of the KANSL1 isoform (Beta=‐27.13, P=0.002) but not with the gene (P>0.05). SNP rs4586175 located in the ROBO3‐ROBO4 locus was significantly associated with symptomatic AD (OR=0.58, CI=0.45‐0.74, P=9.89x10^‐6^) and increased expression of the ROBO3 isoform ENST00000543966 (Beta=2.72, P=0.03). A CpG site cg08770647 located 773 bp from rs4586175 was nominally associated with symptomatic AD (Log2FC=0.06, P=1.52x10^‐4^) and its methylation level was correlated with increased expression of the ROBO3 isoform (Beta=83.26, P=0.01).

**Conclusion:**

Our study suggests that interactions between genetic and epigenetic signatures contribute to cognitive resilience in persons with pathologically confirmed AD. Our findings also provide insight about genetic mechanisms that could be targeted to retard or prevent onset of AD clinical symptoms.

